# Determination of 3-Quinuclidinyl Benzilate in Urine

**DOI:** 10.6028/jres.093.050

**Published:** 1988-06-01

**Authors:** G. D. Byrd, L. T. Sniegoski, E. White

**Affiliations:** Center for Analytical Chemistry, National Bureau of Standards, Gaithersburg, MD 20899

The compound 3-quinuclidinyl benzilate (BZ) is a potent muscarinic cholinergic antagonist that can produce incapacitation at very small doses [1,2]. As such it can be used as a powerful psychochemical warfare agent. In response to the scheduled destruction of U.S. military stockpiles of BZ and the increased potential for worker exposure, our laboratory has developed a specific confirmatory test for human exposure to BZ. The test determines the amount of the parent compound in the urine as well as the two major metabolites, quinuclidinol (Q) and benzilic acid (BA) which are formed by hydrolysis as shown in figure 1. Previous work in our laboratory demonstrated that BA and Q could be determined in urine at their target concentration of 5 ng/mL as based on a proposed acceptable exposure level. The work described here demonstrates the recovery of the parent compound from urine at its target level and the incorporation of this method into an overall test for exposure to BZ.

Only a small percentage of unmetabolized BZ is expected to be found in the urine of exposed persons. Estimates based on the proposed acceptable exposure level require detection limits for BZ in urine of 0.5 ng/mL. Because of the complexity of the urine matrix and the variation of urine from one individual to the next, the measurement of BZ in urine at this level presents a challenging analytical problem. A method using solid phase extraction and isotope dilution GC/MS was developed to measure BZ in urine. In the procedure, a 20 mL urine sample is made basic and the BZ is removed by solid phase extraction onto a C_18_ sample preparation column. The column is washed with water and a 40% acetonitrile solution and the BZ is eluted with methanol. The eluent is blown to dryness and reconstituted in a derivatizing agent to form the trimethylsilyl derivative for analysis by GC/MS. Measurements are performed using single ion monitoring for the fragment ion (C_6_H_5_)_2_CO-TMS^+^ at *m/z* 255 and the analogous ion from the isotopically labeled internal standard (3-quinuclidinyl-^18^O-benzilate-d_5_) at *m/z* 260. We have been able to determine BZ in urine at concentrations less than 0.5 ng/mL. Figure 2 depicts an ion chromatogram showing detection of BZ as its TMS derivative at 0.5 ng/mL.

This method was incorporated into the overall test for exposure to BZ which determines the concentration of BZ, BA, and Q in urine. The test was validated by looking at eight different urine samples which were divided into subsamples, some spiked with known concentrations of the analytes and others left blank. Of the 18 subsamples that were analyzed for BZ, BA, and Q, nine were spiked at or just below the target concentration level, five at approximately 10 times the target level, and four were blank. The subsamples spiked at 10 times the target level were to provide information for urine samples with concentration levels that would be expected in the event of an actual exposure. The blank urine samples provided information regarding background interferences with the test over several different urines. The results of the validation test on these samples are summarized graphically in figure 3.

For all three analytes we considered the measured values close to the spiked levels. Based on the established target levels no false positives occurred for any of the three analytes in any of the four blank urines. For samples spiked near the target levels no measured value was less than 80% of the spiked value. On this basis, no false negatives were apparent. The imprecisions in the method with regard to GC/MS measurement, sample preparation, and urine-to-urine variability were more or less evenly distributed and the imprecision of a single measurement was about 15%.

## Figures and Tables

**Figure 1:**
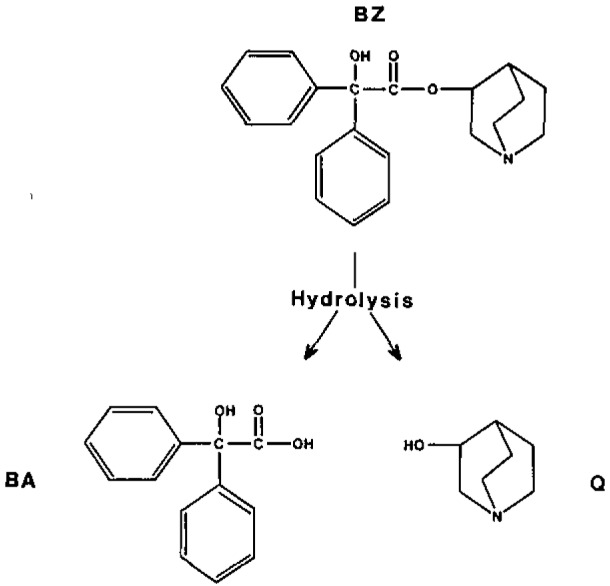
Hydrolysis of BZ.

**Figure 2:**
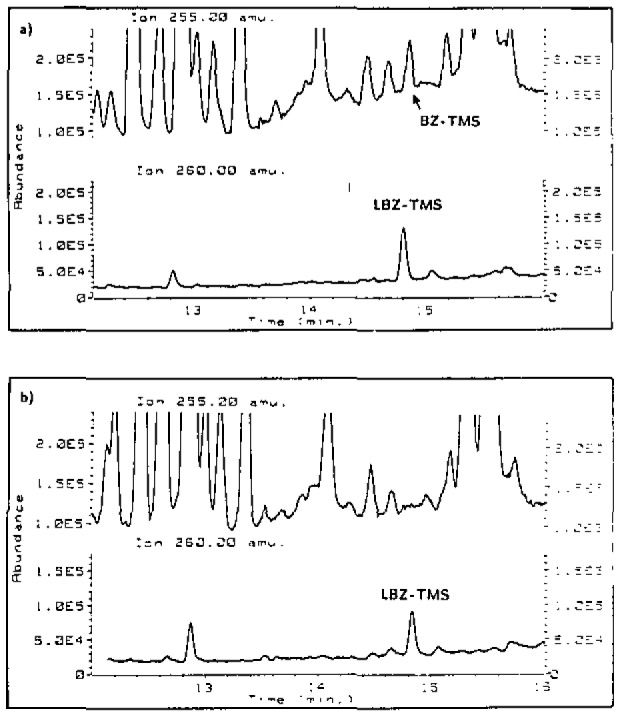
SIM chromatogram of *m/z* 255 and *m/z* 260 from extracts of urine a) spiked with 0.5 ng/mL BZ and b) a urine blank. BZ-TMS is the derivatized BZ and LBZ-TMS is the derivatized labeled internal standard.

**Figure 3:**
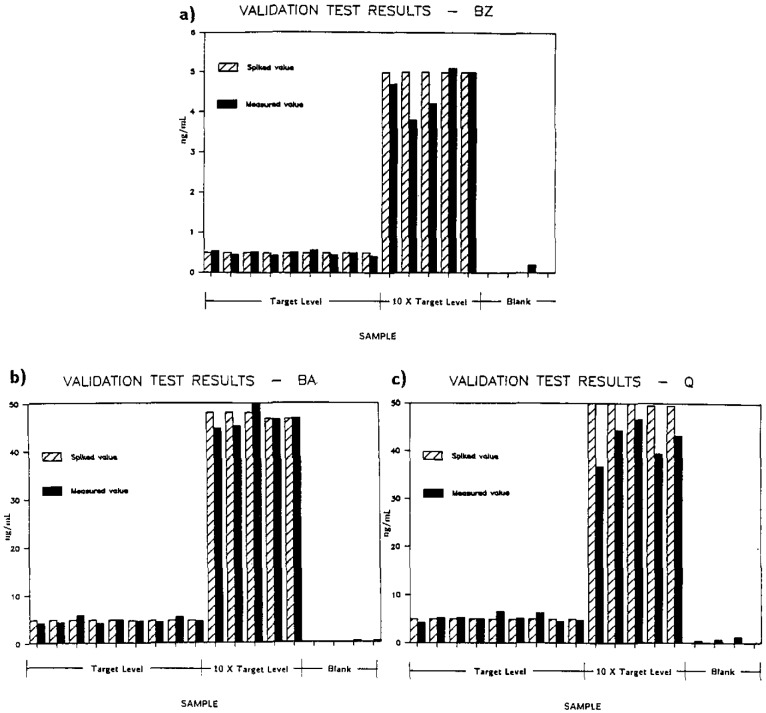
Graphical representation of results from validation test of analytical method for a) BZ, b) BA, and c) Q in urine.
